# Uncovering the Benefits of the Ketamine–Dexmedetomidine Combination for Procedural Sedation during the Italian COVID-19 Pandemic

**DOI:** 10.3390/jcm12093124

**Published:** 2023-04-25

**Authors:** Alessandro Riccardi, Sossio Serra, Fabio De Iaco, Andrea Fabbri, Dana Shiffer, Antonio Voza

**Affiliations:** 1 Emergency Department, Ospedale di Imperia, 18100 Imperia, Italy; 2 Emergency Department, Maurizio Bufalini Hospital, 47522 Cesena, Italy; 3 Emergency Department, Ospedale Maria Vittoria, 10144 Turin, Italy; 4 Emergency Department, AUSL Romagna, Presidio Ospedaliero Morgagni-Pierantoni, 47121 Forlì, Italy; 5 Emergency Department, IRCCS Humanitas Research Hospital, Via Manzoni 56, 20089 Milan, Italy; 6 Department of Biomedical Sciences, Humanitas University, Via Rita Levi Montalcini 4, 20072 Pieve Emanuele, Italy

**Keywords:** sedation of ventilated patients, hemodynamic profile, dexmedetomidine, ketamine

## Abstract

This retrospective observational study evaluated the safety and efficacy of the ketamine and dexmedetomidine combination (keta-dex) compared to ketamine or dexmedetomidine alone for sedation of patients with acute respiratory distress due to COVID-19 pneumonia who require non-invasive ventilation. The following factors were assessed: tolerance to the ventilation, sedation level on the Richmond Agitation-Sedation Scale (RASS), hemodynamic and saturation profile, adverse effects, and discontinuation or mortality during ventilation. The study included 66 patients who underwent sedation for non-invasive ventilation using keta-dex (KETA-DEX group, *n* = 22), ketamine (KET group, *n* = 22), or dexmedetomidine (DEX group, *n* = 22). The DEX group showed a slower sedation rate and a significant reduction in blood pressure compared to the KETA-DEX group (*p* < 0.05). An increase in blood pressure was recorded more frequently in the KET group. No reduction in oxygen saturation and no deaths were observed in any of the groups. None of the patients discontinued ventilation due to intolerance. The mean duration of sedation was 28.12 h. No cases of delirium were observed in any of the groups. Overall, keta-dex was associated with faster sedation rates and better hemodynamic profiles compared to dexmedetomidine alone. Keta-dex is effective and safe for sedation of uncooperative patients undergoing non-invasive ventilation.

## 1. Introduction

The Italian COVID-19 experience highlighted the importance of sedation in critical settings [1]. Agitated patients and elderly patients who are uncooperative toward non-invasive ventilation or CPAP required the use of alternative or less-common techniques to achieve adequate sedation [2,3]. In the context of procedural sedation, propofol [4], midazolam [5] (both of which are GABAergic agonists), or ketamine [6] (an NMDA antagonist, among its various functions) are the most widely used options.

In recent years, there has been an increased use of dexmedetomidine [7,8], a central alpha-2 agonist with an excellent safety profile, but mixed results when used alone for procedural sedation as it lacks analgesic action, has a slow onset, and sometimes exerts hemodynamic effects (bradycardia and hypotension), especially at high doses [9].

Unlike GABA agonists, dexmedetomidine can sedate ventilated patients without causing respiratory depression. Dexmedetomidine also induces natural sleep through direct action on the locus coeruleus, lowering the risk of post-sedation delirium [7,10,11,12]. However, one of the main disadvantages of this drug is its high cost compared to other drugs [13]. During the pandemic, doctors were forced to use ketamine for sedation in ventilated patients due to a drug shortage, and researchers have since investigated the alternatives. Although there are very few studies on the use of dexmedetomidine for the sedation of ventilated patients, its safety has been demonstrated [14,15,16].

The pandemic wave, beginning in March 2020, overwhelmed the available ICU beds in Italy. As a result, different strategies were needed for ventilating patients with respiratory failure due to COVID-19 pneumonia, particularly the elderly and uncooperative patients owing to hypoxic agitation [1]. We believe that dexmedetomidine would be an excellent auxiliary medication to ketamine for sedation in ventilated patients because they have complementary and synergistic effects. Therefore, we conducted this retrospective study to evaluate the safety and efficacy of the ketamine–dexmedetomidine (keta-dex) combination compared to ketamine or dexmedetomidine alone for sedation of agitated patients during non-invasive ventilation.

## 2. Materials and Methods

This retrospective observational, multi-center study included patients sedated with the keta-dex combination and patients sedated with either ketamine or dexmedetomidine during non-invasive ventilation or CPAP due to acute respiratory distress from COVID-19 pneumonia between 1 March and 30 April 2020. Data were obtained from five emergency departments in Italy: Turin, Forli, Cesena, Savona, and Milan. Patients were included in the study if they (1) were 18 years or older, (2) were diagnosed with acute respiratory distress due to COVID-19 pneumonia, (3) required non-invasive ventilation or CPAP, and (4) were sedated with the ketamine–dexmedetomidine combination, ketamine, or dexmedetomidine during non-invasive ventilation. To avoid selection bias, we excluded patients with severe respiratory failure (i.e., P/F < 100 in 100% oxygen therapy).

Patients were grouped based on sedation with the ketamine–dexmedetomidine combination (KETA-DEX group), ketamine (KET group), or dexmedetomidine (DEX group).

The following parameters were evaluated: agitation during ventilation at 30 min and 60 min, tolerance to the ventilation mask or helmet, sedation level, adverse effect of any kind, and discontinuation or mortality during ventilation.

The Richmond Agitation-Sedation Scale (RASS) is a validated assessment tool for determining the sedation depth in critical patients [17]. It is a ten-point scale with positive values for agitation and negative values for reduced arousal levels. The RASS was used to assess sedation levels after 10, 30, and 60 min and every hour thereafter throughout the ventilation period. In addition, age, sex, and vital sign (heart rate, blood pressure, respiratory rate, and oxygen saturation) data were collected.

The centers participating in the study are national reference centers for procedural sedation, where physicians belonging to the Italian society of emergency-urgency medicine (SIMEU) group in sedation and analgesia in emergencies (SAU) work with a sedation registry.

The study did not require an ethics committee evaluation as sedation was determined by an experienced physician for clinical purposes only and data recording was conducted completely anonymously.

Data were analyzed using the statistical functions of Microsoft Excel 365 (2023). Numerical data were expressed as means, and categorical data were expressed as frequencies (percentages). Chi-square tests were performed to examine the relationship between categorical variables. Statistical significance was defined at a *p*-value < 0.05.

## 3. Results

A total of 66 uncooperative patients with acute respiratory distress due to COVID-19 pneumonia who underwent sedation with the ketamine–dexmedetomidine combination, ketamine, or dexmedetomidine for non-invasive ventilation were included in the study (Table 1). The KETA-DEX group included 22 patients (13 females, 9 males, mean age of 78.33 years) who received ketamine (1 mg/kg, followed by 1 mg/kg/h) and dexmedetomidine (0.7 mcg/kg, followed by 0.7 mcg/kg/h). The DEX group included 22 patients (12 females, 10 males, mean age of 77.21 years) who were sedated with dexmedetomidine alone (0.7 mcg/kg in 10 min, followed by 0.7 mcg/kg/h up to 2 mcg/kg/h). In the DEX group, the infusion rate was increased in 47% of patients, reaching the maximum infusion rate of 2 mcg/kg/h in 28% of patients. The KET group consisted of 22 patients (13 females, 9 males, mean age of 79.41 years) who were sedated with ketamine alone (1 mg/kg, followed by 1 mg/kg/h). Standard dosages of the drugs were chosen based on international references.

RASS was evaluated just before sedation, after 10, 30, and 60 min of sedation, and every hour of sedation thereafter. The mean RASS just before sedation was 3 (range 2–4), with all patients with a RASS score of 4 belonging to the ketamine group. In the DEX group, we observed a slower sedation rate, with only 20% of patients adequately sedated at 30 min compared to 99% of patients sedated at 10 min in the KETA-DEX and KET groups. The mean RASS score was −4, with a RASS −1.96 in the DEX group, and a RASS −5 in the KET and KETA-DEX groups. It should be emphasized that the RASS is not designed to assess dissociative sedation, which is a particular result of anesthesia where the patient maintains protective reflexes and vital functions.

We did not observe a reduction in oxygen saturation during sedation in any of the groups. However, we observed a significant reduction in blood pressure in the DEX group (10 out of 12 patients) when compared to the KETA-DEX group (0 out of 22 patients) (X^2^ 16.5, *p* < 0.05). An increase in blood pressure was recorded more frequently in the KET group (10 out of 12 patients) than in the KETA-DEX group (0 out of 22 patients) (X^2^ 12.44, *p* < 0.05).

There was no observed increase in airway secretion, which is a known muscarinic effect of ketamine [6], in either the KET or KETA-DEX groups. However, this effect is most commonly seen in young people and during rapid infusions [6].

All patients were provided with ventilation using masks or helmets. For patients receiving continuous positive airway pressure (CPAP), which included 58 patients in total (20 in the KETA-DEX group, 19 in the KET group, and 19 in the DEX group), the initial positive end-expiratory pressure (PEEP) was set at 7.5, with the fraction of inspired oxygen (FiO2) varying between 50% and 100%. For patients receiving non-invasive ventilation (NIV), which included a total of eight patients (two in the KETA-DEX group, three in the KET group, and three in the DEX group), the mean support pressure was 8 cm H20, with the FiO2 varying between 50% and 100%. There were no reported deaths in any of the three groups, and none of the patients needed to discontinue ventilation due to intolerance. The average duration of sedation was 28.12 h, with the shortest duration being 3 h and the longest being 33 h. Ventilation was discontinued in 12 cases due to non-response, including three in the KETA-DEX group, three in the DEX group, and six in the KET group. Palliative therapy was initiated in these cases. In eight cases, ventilation was discontinued due to an improvement in gas exchange, including four in the KETA-DEX group, three in the DEX group, and one in the KET group. Patients were lost to observation in the remaining cases because they were transferred to other departments. As a result, no cases of delirium were observed in any of the groups.

## 4. Discussion

The results of our study demonstrate that the keta-dex association resulted in good adherence to the use of ventilation masks or helmets and faster sedation rates than dexmedetomidine alone, with a better hemodynamic profile. However, the best results with respect to sedation were observed with ketamine alone. Despite the absence of a label for the use of ketamine for sedation of ventilated patients, its use has become widespread over time [14]. The main limitation of ketamine sedation is the emergence reaction, which is an agitation upon waking that occurs in up to 30% of sedations [18] but rarely requires treatment [19]. In fact, there is no agreement regarding the definition of “emergence reaction”. Many studies include agitation upon waking and psychomimetic symptoms characterized as unpleasant by the patient (vivid dreams, hallucinations) in this category, but such symptoms rarely require specific treatment (usually midazolam, sometimes haloperidol) [6,18,19]. Although psychosis is considered a risk factor for developing these symptoms, a study by Lahti et al. found that the magnitude of ketamine-induced changes in psychotic symptoms between healthy volunteers and schizophrenia patients was comparable, and the dose–response profiles over time were superimposable across the two groups [20]. Although these symptoms do not always require treatment, they may limit the use of ketamine. The rate of “unpleasant” emergence reactions is found to be higher in the emergency department than in the operating room [18,19]. This is because it is now known that a chaotic environment and excessive stimuli (during the onset of sedation and upon waking) are associated with a higher risk of emergence reactions; thus, managing ketamine sedation in a calm environment and, whenever possible, with an informed patient can dramatically transform the experience into a positive one [6,21]. In our case series, we did not observe emergence reactions in the ketamine group as patients sedated with ketamine had a higher RASS score at the beginning, most likely due to more severe pulmonary involvement. The choice of ketamine use in more agitated patients stems from a greater familiarity with ketamine use in patients with severe psychomotor agitation, which explains why ketamine-treated patients have a higher rate of discontinuation to initiate palliation and a lower rate of discontinuation for improvement when compared to dexmedetomidine or the keta-dex combination.

Indeed, strategies to reduce these symptoms have been used, and the combination of ketamine and dexmedetomidine appears to be very beneficial, particularly in the ICU or in unstable patients [22,23,24,25,26,27]. When compared to the other more well-known association between ketamine and propofol (keto-fol), the literature shows better tolerance because it reduces nausea and vomiting, as well as respiratory depression [9,10,11,12,13,14,15,16,18,19,20,21,22,23,26,27], which is already low with keto-fol when compared to propofol [28,29]. Neither ketamine nor dexmedetomidine causes respiratory depression [6,7,8]. In addition, the impact of keta-dex and keto-fol on the cardiovascular system is minimal since both combinations have a synergistic effect on the cardiovascular profile [21,22,23,24,25,26]. Ketamine enhances central catecholaminergic tone with increased levels of circulating norepinephrine and dopamine, whereas dexmedetomidine can cause bradycardia and vasodilation, especially at higher doses. As a result, the effects of the two medications cancel each other out [30,31,32,33].

Our data demonstrate a lower rate of cardiovascular adverse events in the KETA-DEX group than in the DEX group (where more hypotensive events were observed) or the KET group (which had more hypertensive events). The lower dosage of dexmedetomidine in KETA-DEX group probably explains this observation. Indeed, in the DEX group, infusion augmentation occurred in 47% of cases, and the maximum rate of 2 mcg/kg/h was seen in 28%; this group also displays the highest prevalence of adverse events (hypotension). Another advantage observed in the KETA-DEX group (versus sedation with dexmedetomidine alone) was the more rapid onset of sedation.

This combination is especially beneficial in critically ill patients, especially those with respiratory failure, because it improves hemodynamic balance and has a lower rate of respiratory depression than propofol or keto-fol [7,8]. Furthermore, the addition of ketamine to dexmedetomidine compensates for the disadvantages of using dexmedetomidine alone, particularly its slow action [9]. Dexmedetomidine has been shown in several double-blind, randomized trials and a meta-analysis [23] to significantly reduce the rate of severe emergence agitation in ketamine-treated patients [9]. Since ketamine-induced reuptake of neuronal catecholamine induces a catecholaminergic state that increases norepinephrine, which contributes to ketamine-induced agitation, the addition of a central alpha-2 agonist, such as dexmedetomidine, attenuates this effect, thus reducing the complications normally observed when ketamine is used alone [33,34,35,36]. A variety of therapeutic approaches are available, including administering 1 mg/kg of ketamine with 0.5–1 mcg/kg of dexmedetomidine; if necessary, the latter could be infused at 1–2 mcg/kg/h.

The absence of oxygen desaturation during sedation with dexmedetomidine, ketamine, or keta-dex confirms the excellent safety profile with regard to the respiratory function of the two drugs and their combination.

There are several limitations in our study. First, the retrospective nature of the study prevents accurate evaluations. However, our observations confirm what has been described in the literature, and the observed benefits are compatible with the pharmacological effects of the two molecules. Second, our sample population is limited, and prospective studies will be able to define the benefits of the combination more definitively. Finally, our very select case series, namely elderly patients with COVID-19-related respiratory failure, would, in an emergency setting such as the first pandemic wave, possibly limit the validity of our observations.

Nevertheless, in our opinion and according to our study, the keta-dex combination, which has received little attention in the literature, has undeniable efficacy and safety benefits that merit further investigation with dedicated studies.

## 5. Conclusions

The study suggests that the keta-dex combination is associated with good adherence to the use of ventilation masks or helmets and faster sedation rates than dexmedetomidine alone, with a better hemodynamic profile. Furthermore, though ketamine alone resulted in the best sedation outcomes, its main limitation is the emergence reaction risk. Therefore, strategies to reduce these symptoms, such as combining ketamine and dexmedetomidine, have been shown to be beneficial, particularly in the ICU or in unstable patients. The observations of our study confirm the efficacy and safety profile of the ketamine–dexmedetomidine combination.

## Figures and Tables

**Table 1:** Demographics, baseline clinical characteristics, and results obtained in the 3 groups.

	KETA-DEX Group	KET Group	DEX Group
Patients (*n*)	22	22	22
Male % (*n*)	40.9 (9)	40.9 (9)	45.5 (10)
Mean age (years)	78.33 ± 8.72	79.41 ± 10.76	77.21 ± 12.33
Hypertension % (*n*)	86.4 (19)	68.2 (15)	72.7 (16)
Diabetes % (*n*)	36.3 (8)	27.3 (6)	22.7 (5)
Neoplasm history % (*n*)	22.7 (5)	27.3 (6)	31.8 (7)
Heart failure % (*n*)	40.9 (9)	27.7 (6)	36.3 (8)
Mean P/F before sedation	145.45 ± 23.46	138.59 ± 20.67	147.45 ± 26.25
Mean RASS	−5 ± 0	−5 ± 0	−1.96 ± 0.21
10′ RASS	−5 ± 0	−5 ± 0	0
30′ RASS	−5 ± 0	−5 ± 0	−0.82 ± 0.39
60′ RASS	−5 ± 0	−5 ± 0	−1.81 ± 0.39
Desaturation (*n*)	0	0	0
Change in blood pressure (*n*)	0	10 (hypertension)	10 (hypotension)

Data are expressed as absolute numbers (*n*), percentages (%), or mean ± standard deviation.

## Data Availability

The data presented in this study are available on request from the corresponding author.
